# Evaluation of the Differences in the Expression of Biogenic Amine-Related mRNAs and Proteins in Endometrioid Endometrial Cancer

**DOI:** 10.3390/jcm10214872

**Published:** 2021-10-22

**Authors:** Michał Czerwiński, Anna Bednarska-Czerwińska, Nikola Zmarzły, Dariusz Boroń, Marcin Oplawski, Beniamin Oskar Grabarek

**Affiliations:** 1 American Medical Clinic, 40-600 Katowice, Poland; 2 Gyncentrum Fertility Clinic, 40-121 Katowice, Poland; czerwinskaa002@gmail.com; 3 Department of Histology, Cytophysiology and Embryology, Faculty of Medicine, University of Technology in Katowice, 41-800 Zabrze, Poland; nikola.zmarzly@gmail.com (N.Z.); dariusz@boron.pl (D.B.); bgrabarek7@gmail.com (B.O.G.); 4 Department of Gynecology and Obstetrics with Gynecologic Oncology, Ludwik Rydygier Memorial Specialized Hospital, 31-826 Kraków, Poland; marcin.oplawski@gmail.com; 5 Departament of Gynecology and Obstetrics, TOMMED Specjalisci od Zdrowia, Fredry 22, 40-662 Katowice, Poland

**Keywords:** endometrial cancer, biogenic amines, histamine receptors, dopamine receptors, expression profile

## Abstract

Biogenic amines, such as adrenaline, noradrenaline, histamine, dopamine, and serotonin are important neurotransmitters that also regulate cell viability. Their detection and analysis are helpful in the diagnosis of many diseases, including cancer. The aim of this study was to determine the expression profile of the biogenic amine-related genes and proteins in endometrioid endometrial cancer compared to the control group. The material consisted of endometrial tissue samples and whole blood collected from 30 endometrioid endometrial cancer patients and 30 cancer-free patients. The gene expression was determined by the mRNA microarrays and validated by qRT-PCR. Protein levels were determined in the serum by the enzyme-linked immunosorbent assay (ELISA). Overexpression of histamine H1–H3 receptors and early growth response 1 and silencing of calmodulin, the histamine H4 receptor, and the dopamine D5 receptor have been reported in endometrioid endometrial cancer. The obtained results indicate disturbances in the signaling activated by histamine and dopamine receptors, which could potentially contribute to the progression of endometrioid endometrial cancer.

## 1. Introduction

Biogenic amines (BAs) are low-molecular-weight compounds formed mainly by the decarboxylation of amino acids. They are widely described in the context of food, as they can be produced by its microbiome, which affects the quality of the product [1]. Too high concentrations of BAs can also cause negative health effects such as nausea, headaches, vomiting, hypertension, or diarrhea [2]. Interestingly, at the cellular level, they affect cell viability and participate in the synthesis of proteins and in the course of many metabolic processes [3]. In addition to adrenaline and noradrenaline, histamine, dopamine, and serotonin are also important biogenic amine neurotransmitters [4]. Moreover, they play an important role in the immune response, thermoregulation, gut and cardiovascular function, and sleep and mood regulation. It is believed that the detection of BAs may be useful in the diagnosis and therapy of cardiovascular and neuroendocrine diseases, as well as cancer [5].

Histamine and its four receptors (H1–H4 encoded by the *HRH1*–*HRH4* genes) are mainly involved in inflammatory reactions and may affect the proliferation and angiogenesis of neoplastic cells, favoring the initiation and further progression of cancer [6]. Changes in their activity have been observed in gastric cancer [7], pancreatic cancer [8], colorectal cancer [9], hepatocellular carcinoma [10], and cholangiocarcinoma [11]. The deregulation of the dopaminergic system, the activity that depends on the dopamine D1–D5 receptors (DRD1–DRD5), may also play a role in the proliferation, apoptosis, and migration of breast and colon cancers [12], ovarian cancer [13], and gastric cancer [14].

Endometrial cancer (EC) is one of the most frequently diagnosed gynecological cancers in the world. In 2020, it was the sixth most common cancer in women overall [15]. Most cases include endometrioid endometrial cancer and affect postmenopausal women [16]. However, it is worth mentioning that up to 25% of cases are diagnosed in premenopausal women [17]. The risk factors for EC include diabetes, unopposed estrogen therapy, obesity, the use of tamoxifen, nulliparity, and polycystic ovary syndrome (PCOS) [18]. In recent years, attempts have been made to implement new classification methods to improve the precision of diagnosis and treatment [19]; however, many aspects related to the initiation and progression of endometrial cancer remain unclear.

The aim of this study was to determine the expression profile of biogenic amine-related genes and proteins in endometrioid endometrial cancer compared to the control group.

## 2. Materials and Methods

This study was approved by the Bioethical Committee operating at the Regional Medical Chamber in Kraków (185/KBL/OIL/202 and 186/KBL/OIL/2020). All procedures involving human participants were performed in accordance with the guidelines of the 2013 Declaration of Helsinki. Data confidentiality and patient anonymity were maintained at all times. Written informed consent was obtained from all study participants.

### 2.1. Patients

The study included endometrial tissue and blood samples collected from 60 women who qualified for a hysterectomy and were treated at the Department of Gynecology and Obstetrics with Gynecologic Oncology at the Ludwik Rydygier Memorial Specialized Hospital. The study group consisted of 30 patients with endometrioid endometrial cancer diagnosed on the basis of histopathological examination. The collected EC samples were further divided according to the degree of histological differentiation: G1, 15 cases; G2, 8 cases; G3, 7 cases. The control group involved 30 patients without any neoplastic changes.

The exclusion criteria from the study group comprised the diagnosis of a cancer other than endometrioid endometrial cancer coexisting with cervical cancer, endometriosis, or adenomyosis; the use of hormone therapy 24 months before the surgery; a history of other cancer types; and extreme obesity (BMI > 40). The patients enrolled in the study were over 45 years old and past childbearing age.

Endometrial tissue samples were placed in Eppendorf tubes with Allprotect Tissue Reagent (Qiagen, Cat. No./ID: 76405), while whole blood was collected using PAXgene Blood RNA Tubes. The samples were then stored according to the manufacturers’ instructions until molecular analysis.

### 2.2. RNA Isolation

Total RNA extraction from endometrial tissue samples was carried out with TRIzol reagent (Invitrogen Life Technologies, Carlsbad, CA, USA, Cat. No. 15596026) as recommended by the manufacturer. The PAXgene Blood RNA Kit (Qiagen, Cat. No./ID: 762174) was used to extract RNA from the whole blood.

The obtained extracts were assessed qualitatively and quantitatively by agarose electrophoresis and spectrophotometry. A 260/280 ratio of 1.8–2.0 allowed us to qualify the extract for a microarray analysis.

### 2.3. mRNA Microarrays

The biogenic amine-related gene expression profile was determined using HG-U133A 2.0 oligonucleotide microarrays (Affymetrix, Santa Clara, CA, USA), the GeneChip™ 3′IVT PLUS Reagent Kit (ThermoFisher, Waltham, MA, USA, Cat. No. 902416), and the GeneChip™ HT 3′IVT PLUS Reagent Kit (ThermoFisher, Waltham, MA, USA, Cat. No. 902417).

The names of the probes and their identification numbers were obtained by entering the phrase “biogenic amine” in the Affymetrix NetAffx™ Analysis Center database (http://www.affymetrix.com/analysis/index.affx; accessed on 1 July 2021). Gene Array Scanner (Agilent Technologies, Santa Clara, CA, USA) was used to measure the fluorescence intensity.

### 2.4. Real-Time Quantitative Reverse Transcription PCR

Real-Time Quantitative Reverse Transcription PCR (qRT-PCR) was carried out to validate the microarray results. The expression profile of *HRH1*, *HRH2*, *HRH3*, *HRH4*, *CALM2*, *DRD5*, *EGR1*, and *ICAM1* was determined with SensiFast SYBR No-ROX One-Step Kit (Bioline, London, UK). β-actin (ACTB) was used as the endogenous control. The reaction thermal profile consisted of: reverse transcription (45 °C, 10 min), polymerase activation (95 °C, 2 min), and 40 cycles including denaturation (95 °C, 5 s), annealing (60 °C, 10 s), and elongation (72 °C, 5 s).

### 2.5. ELISA

The level of the HRH1, HRH2, HRH3, HRH4, CALM2, DRD5, EGR1, and ICAM1 proteins was determined using the following ELISA kits: Human HRH1 Kit (MyBioSource, Inc., San Diego, CA, USA, Cat. No. MBS2602695), Human HRH2 Kit (MyBioSource, Inc., San Diego, CA, USA, Cat. No. MBS265945), Human HRH3 Kit (MyBioSource, Inc., San Diego, CA, USA, Cat. No. MBS450109), Human HRH4 Kit (MyBioSource, Inc., San Diego, CA, USA, Cat. No. MBS2023167), Calmodulin Kit (MyBioSource, Inc., San Diego, CA, USA, Cat. No. MBS455890), Human Dopamine Kit (MyBioSource, Inc., San Diego, CA, USA, Cat. No. MBS264001), Egr-1 Kit (MyBioSource, Inc., San Diego, CA, USA, Cat. No. MBS3805004), and ICAM1 Kit (Abcam, Cambridge, MA, USA, Cat. No. ab174445). The generated standard curve allowed us to assess the concentration of the studied proteins in the patients’ serum.

### 2.6. Statistical Analysis

The statistical analysis was carried out using the Transcriptome Analysis Console Software (Thermo Fisher Scientific, Waltham, MA, USA) and the Statistica 13.0 PL (Kraków, Poland). The ANOVA and Tukey’s post hoc test were performed (*p* < 0.05). The changes in the gene expression are presented as a fold change (FC).

## 3. Results

### 3.1. Biogenic Amine-Related Genes Expression Profile in Endometrial Tissues Determined by Microarrays and RT-qPCR

**Figure 1:**
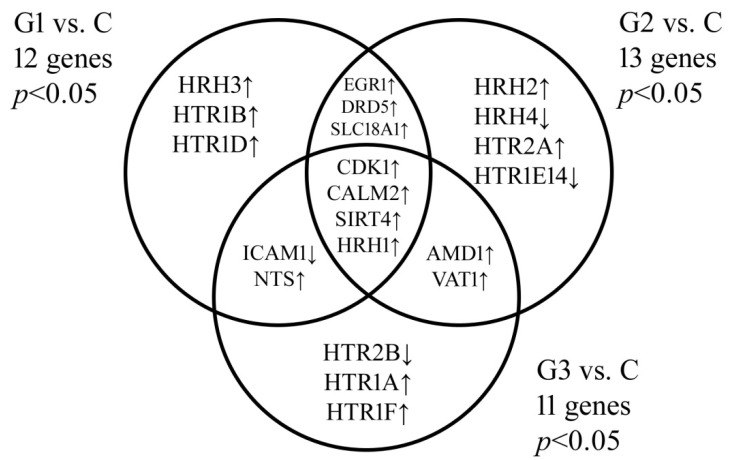
Venn diagram of microarray results showing genes differentiating endometrial cancer from the control group. C, control group; G, endometrial cancer grade; *p* < 0.05 vs. the C group.

One-way ANOVA with the Benjamini–Hochberg correction revealed that out of 292 biogenic amine-related mRNAs, the expression of 33 mRNAs representing 21 genes was significantly altered in endometrial cancer tissue samples compared to the control group. Tukey’s post hoc test and the subsequent construction of the Venn diagram allowed us to visualize which genes are characteristic of a given grade or common to several groups (Figure 1).

The results showed that the expression of *CDK1*, *CALM2,* and *SIRT4* was significantly increased in all grades of endometrial cancer compared to the control group. The *HRH1* and *HRH2* genes were characteristic of G1 and G2 EC, respectively. *HRH1* was also common to the G2 and G3 samples. An increased level was also noted for *HRH3*, while *HRH4* showed a decrease in expression compared to the control group. In addition, a significant increase in the levels of *EGR1*, *DRD5*, and *SLC18A1* was revealed. In the case of G3 cancer, a decrease in *HTR2B* expression and the overexpression of *HTR1A* and *HTR1F* were also noted. Moreover, it was observed that *ICAM1* and *NTS* are common to G1 and G3 cancer.

In the next step, the expression profile of *HRH1*, *HRH2*, *HRH3*, *HRH4*, *CALM2*, *DRD5*, *EGR1*, and *ICAM1* in endometrial tissues was assessed with qRT-PCR. Table 1 summarizes the results of the microarray analysis and qRT-PCR (*p* < 0.05). The following values are shown as a fold change (FC).

The results obtained in the microarray experiment were successfully validated. A decrease in expression regardless of cancer grade was observed for *CALM2*, *DRD5*, *ICAM1*, while *EGR1*, *HRH1*, *HRH2*, *HRH3* were overexpressed. There was also a de-crease in the level of HRH4 in G1 and G2 EC and an increase in G3 cancer.

### 3.2. Level of Biogenic Amine-Related Proteins in the Serum of Patients Determined by ELISA

The expression of the HRH1, HRH2, HRH3, HRH4, CALM2, DRD5, EGR1, ICAM1 proteins was assessed in the serum of EC patients and in the control group with ELISA (Table 2).

The analysis showed that the expression of HRH1, HRH2, and HRH3 increased with the progression of endometrial cancer. The observed overexpression was con-sistent with the results at the gene level. Similarly, for DRD5 and ICAM1 there was a consistent decrease in the protein levels. Slight discrepancies were observed for CALM2 in G1 cancer and for HRH4 in G3 cancer.

## 4. Discussion

According to the WHO, cancer is one of the leading causes of death worldwide [20]. In 2020, approximately 20 million new cases were diagnosed worldwide, almost half of which were in women [21]. Moreover, premature deaths due to cancer have led to the search for new diagnostic methods or potential therapies. However, the complexity of the processes and the relationships taking place within the tumor make it difficult [22]. The detection of biogenic amines or their metabolites may be a promising solution. They play important functions in the body, including signal transduction or modulation of the immune response, and disturbances in their concentration indicate a possible pathological process [5].

The usefulness of determining the level of biogenic amines has been described in the case of the diagnosis of Parkinson’s disease [23] and central nervous system infections such as bacterial and viral meningitis [24], the pathophysiology of schizophrenia spectrum disorders and the impact of antipsychotic treatment [25], the importance of vaginal bacteria in bacterial vaginosis [26], a better understanding of inflammatory bowel diseases [27], and the diagnosis of early ischemic stroke [25]. It is also helpful in the early diagnosis of neuroendocrine tumors [28], in the development of new methods of examining neoplastic cells [29], or in new therapies targeting biogenic amines, including those based on the use of antihistamines [30].

Histamine is synthesized by L-histidine decarboxylase and, through para or autocrine action, it participates in the processes promoting tumor growth, such as the regulation of the immune response, and the proliferation, angiogenesis, differentiation, apoptosis, and the migration of cancer cells [31]. Its pleiotropic action is possible due to its binding to H1–H4 receptors, the expression of which varies depending on the type of tissue [32]. H1 receptor activation is associated with the further activation of phospholipase C and an increase in intracellular Ca^2+^ levels. Their effect may be regulated by calmodulin and encoded by the *CALM* gene [33]. Fernández-Nogueira et al. reported an H1 receptor overexpression in basal and human epidermal growth factor receptor 2 (HER2)-enriched breast cancers, which correlated with a worse prognosis. They proposed a therapeutic approach based on the use of terfenadine—an antagonist of the histamine H1 receptor—as an inhibition of the migration of basal breast cancer cells, and an induction of their apoptosis was noticed [34]. Zhao et al. reported similar observations for hepatocellular carcinoma. An increased expression of the H1 receptor led to an excessive cell cycle and the suppression of the apoptosis, which promoted metastasis. The use of terfenadine on the HCC xenograft nude mice model inhibited tumor growth [35]. In turn, Matsumoto et al. reported overexpression of the H1 receptor in cisplatin-resistant HeLa cells. The use of receptor antagonists, including cloperastine, allowed for the selective killing of tumor cells [36]. Desloratadine and loratadine may also prove promising in cancer therapy, but more research is needed [31]. These observations may also be helpful in developing new therapeutic targets in endometrial cancer. Our study revealed H1 receptor overexpression in EC patients both at the gene and protein levels. Moreover, this expression increased with the cancer grade, which may suggest that excessive activity of the H1 receptor is involved in processes related to cancer progression, including proliferation and apoptosis, such as in the previously mentioned hepatocellular carcinoma. Thus, it would be beneficial to study the effect of targeting the H1 receptor on the development of endometrial cancer.

The histamine H2 receptor activation is associated with an increase in intracellular cyclic adenosine monophosphate levels. In addition, effects induced in many biological processes, for example, the immune response, are opposite to that of the H1 receptor [37]. Interestingly, Gao et al. noted that colon cancer patients with H2 receptor overexpression demonstrated better overall survival [38]. In the case of the H3 receptor, its increased level has been reported in breast cancer, suggesting its participation in the regulation of tumor growth [39]. Chen et al. observed H3 receptor overexpression in prostate cancer and proposed its inhibition as a promising approach in the treatment of this cancer [40]. The H4 receptor may also be a potential therapeutic target, due to its involvement in antitumor immunity, as shown in the triple-negative breast cancer model [41]. Decreased levels of this receptor have been observed in colorectal cancer [42] and in oral tongue squamous cell carcinoma [43]. On the other hand, the H4 receptor overexpression reduced the proliferation of cholangiocarcinoma [44] and esophageal squamous cell carcinoma [45]. In the case of endometrial cancer, Wang et al. recorded the expression of H1 and H2 receptors in the HEC-1 cell line, while H3 and H4 receptors were almost undetectable. During the analysis, the highest expression was found for the H1 receptor [46]. This is mostly consistent with the results obtained in our study. We observed an overexpression of the H1, H2, and H3 receptors in patients with endometrial cancer compared to the control group. In addition, the expression of the H4 receptor was significantly decreased. This indicates that the activity of the histaminergic pathway changes in the course of endometrial cancer, suggesting its involvement in tumor growth. Restoring the normal expression of the histamine receptors may be a promising target in the treatment of endometrial cancer.

In our study, we also noted a reduction in the expression of calmodulin and the intercellular adhesion molecule 1 (ICAM1), and an overexpression of the early growth response 1 (EGR1). As previously mentioned, the H1 receptor activation is associated with the activation of calcium-dependent signaling, which may be mediated by calmodulin. A decrease in its level in patients with endometrial cancer may suggest possible disturbances in signaling involving this protein or indicate a different way of signal transduction in endometrial cancer cells. In the case of EGR1, the observed overexpression may be associated with increased activity of the H1 receptor. Hao et al. observed that histamine induces EGR1 expression in primary human aortic endothelial cells and it depends on H1 receptor activation [47]. Moreover, the EGR1 may increase the expression of genes encoding catecholamine biosynthesis enzymes [48]. Yoon et al. described the possible involvement of EGR1 in tumor growth and colorectal cancer metastasis by promoting angiogenesis [49]. Our results are also consistent with previous studies assessing the level of EGR1 in endometrial cancer cells [50,51].

Dopamine, which belongs to the catecholamines, acts as a neurotransmitter and as a hormone. It acts by binding with the D1–D5 receptors, participating in the regulation of the immune response and in the functioning of the central nervous system, but also in oncogenesis [52]. However, the effect triggered by the activation of the dopamine receptors depends on the type of cancer as well as the biological context [53]. In this study, decreased expression of the dopamine D5 receptor was observed at both the gene and the protein levels. Interestingly, Prabhu et al. reported an overexpression of the D2 receptor in endometrial cancer, which is an antagonist of the D5 receptor [54]. On the other hand, an overexpression of the D5 receptor has been shown in hepatocellular carcinoma, gastric cancer, and glioblastoma, which contributed to their development [55]. The results obtained in this study indicate a possible disturbance in the signal transduction with the participation of the dopamine D5 receptor.

The detection and analysis of the concentration of biogenic amines can be beneficial in diagnostics because cells secrete molecules at the early stages of the disease. However, given the fact that molecular changes precede phenotypic changes, it may be valuable to analyze potential disorders at the gene level. Our study showed that in endometrial cancer, the greatest disturbances could be observed in the case of the histamine receptors. Excessive expression of the H1–H3 receptors and the EGR1, as well as the silencing of the H4 receptor and calmodulin, may suggest their potential relationship with endometrial cancer progression. A decreased expression of the dopamine D5 receptor may also play an important role, but this requires further research. In the case of adrenaline, noradrenaline, and serotonin receptors, there were no significant changes compared to the normal endometrium.

The present study revealed the expression profile of the biogenic amine-associated genes in endometrial cancer, which was then validated at the protein level. The knowledge on this subject in EC has been limited; therefore, the obtained results constitute a valuable addition. It is worth mentioning, however, that the small group of patients was undoubtedly a limitation of this study. In addition, our attention was focused on endometrioid endometrial cancer, which affects the vast majority of patients, but studies on other EC types may further expand the knowledge about this cancer. In further stages of research, it would be beneficial to follow the signaling pathways activated by the histamine and dopamine receptors. Considering that disturbances in the functioning of the histaminergic and dopaminergic systems can affect cellular processes, including proliferation and apoptosis, which are important for tumor progression, it would be promising to follow them more closely in the course of endometrial cancer using cell cultures. It would also be interesting to study the effect of the antagonists of these amines, which could allow for the selection of new therapeutic targets in endometrial cancer.

## Figures and Tables

**Table 1:** Expression profile of selected biogenic amine-related genes in endometrial tissue samples determined by microarrays and qRT-PCR (*p* < 0.05).

ID	mRNA	Microarray			qRT-PCR		
		G1 vs. C	G2 vs. C	G3 vs. C	G1 vs. C	G2 vs. C	G3 vs. C
207243_s_at	*CALM2*	−9.69	−10.66	−14.01	−11.23	−10.64	−15.88
208486_at	*DRD5*	−4.25	−3.69	−4.87	−4.65	−4.01	−4.30
201693_s_at	*EGR1*	11.25	10.58	14.22	10.52	10.63	13.25
201694_s_at	*EGR1*	11.26	10.41	14.03			
202637_s_at	*ICAM1*	−8.66	−17.25	−14.66	−8.55	−18.41	−12.63
202638_s_at	*ICAM1*	−8.41	−17.77	−14.69			
215485_s_at	*ICAM1*	−8.69	−18.03	−15.01			
205579_at	*HRH1*	1.98	2.14	2.75	1.98	2.14	2.75
205580_s_at	*HRH1*	1.99	2.47	2.81			
220805_at	*HRH2*	3.11	3.02	2.14	3.44	2.65	2.14
220447_at	*HRH3*	2.74	6.14	5.75	2.74	7.01	5.75
221663_x_at	*HRH3*	2.66	6.74	5.98			
221169_s_at	*HRH4*	−2.14	−3.66	2.88	−2.14	−3.66	2.88
221170_at	*HRH4*	−2.36	−3.41	2.54			

ID number of the probe; C, control group; G, endometrial cancer grade.

**Table 2:** Expression profile of biogenic amine-related proteins in serum in the study and in the control groups (*p* < 0.05).

Proteins	Group			
	C	G1	G2	G3
CALM2	2047.55 ± 15.25	2365.14 ± 8.96	524.06 ± 4.58	109.99 ± 2.74
DRD5	4.58 ± 0.85	2.01 ± 0.74	0.89 ± 0.11	0.66 ± 0.36
EGR1	365.54 ± 7.77	1254.01 ± 2.36	541.36 ± 3.41	1698.36 ± 11.25
ICAM1	1854.63 ± 3.14	654.01 ± 2.11	402.01 ± 4.08	107.33 ± 3.54
HRH1	4.44 ± 0.48	9.58 ± 1.22	10.44 ± 0.94	16.74 ± 1.07
HRH2	1.47 ± 0.26	2.44 ± 0.55	4.66 ± 1.04	8.14 ± 1.58
HRH3	2.18 ± 0.73	3.44 ± 0.26	4.69 ± 0.37	7.11 ± 0.12
HRH4	9.12 ± 0.25	7.64 ± 0.49	3.14 ± 0.19	1.01 ± 0.34

C, control group; G, endometrial cancer grade; ELISA—enzyme-linked immunosorbent assay.

## Data Availability

The data used to support the findings of this study are included in the article. The data will not be shared due to third-party rights and commercial confidentiality.
